# Burst of Young Retrogenes and Independent Retrogene Formation in Mammals

**DOI:** 10.1371/journal.pone.0005040

**Published:** 2009-03-27

**Authors:** Deng Pan, Liqing Zhang

**Affiliations:** 1 Department of Computer Science, Virginia Tech, Blacksburg, Virginia, United States of America; 2 Program in Genetics, Bioinformatics, and Computational Biology, Virginia Tech, Blacksburg, Virginia, United States of America; University of Texas Arlington, United States of America

## Abstract

Retroposition and retrogenes gain increasing attention as recent studies show that they play an important role in human new gene formation. Here we examined the patterns of retrogene distribution in 8 mammalian genomes using 4 non-mammalian genomes as a contrast. There has been a burst of young retrogenes not only in primate lineages as suggested in a recent study, but also in other mammalian lineages. In mammals, most of the retrofamilies (the gene families that have retrogenes) are shared between species. In these shared retrofamilies, 14%–18% of functional retrogenes may have originated independently in multiple mammalian species. Notably, in the independently originated retrogenes, there is an enrichment of ribosome related gene function. In sharp contrast, none of these patterns hold in non-mammals. Our results suggest that the recruitment of the specific L1 retrotransposons in mammals might have been an important evolutionary event for the split of mammals and non-mammals and retroposition continues to be an important active process in shaping the dynamics of mammalian genomes, as compared to being rather inert in non-mammals.

## Introduction

Retroposition, a major mechanism of gene duplication, can provide raw materials for the generation of new gene functions [1] and is an important process shaping the evolution of genomes [2]. Retroposition is a process in which mRNAs are reverse-transcribed into DNAs and then insert back into a new position on the genome. Retroposed copies (retrocopies) lack many of their parental genes' genetic features, such as introns and regulatory elements. Most of retrocopies have turned into pseudogenes (also known as processed pseudogenes) in mammals [3], [4], [5], [6], and some of them may happen to recruit upstream regulatory elements and become functional [2], [7] (hereafter called retrogene).

As the survival rate of retrocopies is low, retrocopies have long been viewed as evolutionary dead ends with little functional significance [8]. Recently, a significant number of functional retrocopies (i.e. retrogenes) have been identified in the genomes of mammals and insects [9], [10], [11], [12], , which raised the interest in studying the functional contribution from retroposition. Many interesting features of retrogenes have been unveiled. For example, it has been shown that retrogenes are not randomly located on chromosomes and genes are more likely to be retroposed bidirectionally into and out-of the X chromosome in mammals [10]. Retrogenes seem to show biased functions with the majority of them specifically expressed in testis and closely related to male functions [11], [12], [13].

Marques et al. [11] found a burst of retroposition in human that gave rise to many young retrogenes and thus claimed that retrogenes significantly contribute to the formation of new human genes. The importance of retrogenes in human suggests yet another exciting viewpoint of human origin. However, our recent study [14] shows that retroposition seems to have generated more duplicated genes in mouse than in human. This led us to conclude that retroposition is at least as important in mouse as in human and to speculate that the rapid emergence of young retrogenes might be a common phenomenon in mammals, rather than a unique one in human.

Retroposition is believed to be driven by the enzymatic machinery of LINE1 (Long Interspersed Nucleotide Element 1, L1) [15]. L1s are widely present in mammals and account for up to about 25% of the genomes [16], [17]. Only in some rare cases, L1s are reported to be recently extinct in some mammalian species [18]. The rates of L1 retrotransposon evolution differ in some mammals [19]. However, since only a few closely related L1 lineages are active in mammals [16], [18], the homology between the L1s in different mammalian species may lead to similar enzymatic activity of retrotransposases that are essential for producing retrocopies. Thus, the overall pattern of retroposition dynamics might be similar in most mammals. With the sequenced mammalian genomes, we can test whether the burst of young retrogenes observed in Marques et al. [11] is actually a shared phenomenon among the mammals.

If the burst of young retrogenes is a common phenomenon in mammals, we can also infer that many retrogenes might have emerged independently in different mammalian lineages. It is generally accepted that the prevalence of a certain kind of heritable retrocopy is accompanied with the high germ line expression of the corresponding mRNAs [5], [6]. Highly expressed germ line genes, such as ribosomal proteins, cyclophilin, keratin, GAPDH, and cytochrome C, are the major categories of human processed pseudogenes. Among these processed pseudogenes, ribosomal proteins account for almost one-fifth of the total [5], [20], [21]. Assuming that both the categories of highly expressed germline genes and retroposition dynamics are similar in different mammals, we expect that there might have been many instances of independent retropositions in the same gene families in multiple mammalian lineages.

To examine these expectations, we analyzed the retrogenes in 8 mammalian genomes using 4 non-mammalian genomes as a contrast. Our results show that the patterns of retrogene origination are similar and rapid emergence of young retrogenes is observed in all the studied mammals. Moreover, many retrogenes were generated independently in multiple mammals. Retrogenes show a drastic different dynamic pattern in non-mammals. Clearly retrogenes have played an important role in the evolution of mammals.

## Results

### Retrogene Datasets

Retrocopies can be classified into different categories. At the sequence level, a retrocopy can be either intact (having complete open reading frame with no frameshift mutations and no premature stop codons as compared to its parental gene) or broken (processed pseudogenes). At the expression level, a retrocopy can be either expressed or non-expressed. For example, up to 20% of pseudogenes (including broken retrocopies) are expressed and maybe have functions [22]. To avoid misunderstanding, we define a *retrogene* as an “intact” retrocopy that has transcriptional evidence. Our definition of retrogene is compatible with previous studies [9], [10], [11], [12], [13], and is consistent with the updated version of the definition of a *gene* [22], i.e., a gene should have some sequence structures and encode potentially functional products.

Since not all the species that we surveyed have enough expression evidence for retrogenes, we took steps to ensure both high data quality (i.e. to minimize the influence of pseudogenes) and sufficient number of genes. The detailed data quality control procedures are presented in the Online Supplement File 1. Briefly, in human, mouse, and fruitfly, all the retrogenes obtained completely conform to our retrogene definition, thus the datasets of these three species are of very high quality. In rat, dog, cow, and zebrafish, we had to include some predicted genes to maintain enough candidate retrogenes, despite which, the most conservative estimate of the probability of a retrocopy being a true retrogene in these species is still as high as 75%–90%. In chimp, macaca, opossum, chicken, and anopheles, we required all parental-retrogene pairs in the datasets to have 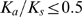, a computational criterion that has been previously validated for ensuring the functionality of retrogenes [9], [10], [11], [13]. For these species, we estimated that about 40% to 70% retrocopies included in the datasets are most likely functional retrogenes.

Summary statistics of retrogenes are shown in Table 1 (see Table S1 for a full list of retrogenes). The numbers of retrogenes are generally similar to those of retrogenes in previously studied species, such as human, mouse and rat. For fruitfly, our observed number is about twice as much as Bai et al. [13]'s observation. The difference is because they limited the number of retrogene through a likelihood ratio test, however, we think it is too conservative (see Text S1 for a detailed discussion). Maybe due to the low annotation quality, the number of retrogenes in dog and Anopheles are a little lower than their other related species. For clarity, we denote the gene family that has at least one retrogene as *retrofamily*. Table 1 shows that the number of retrogenes and the number of retrofamilies are approximately equal in all the species, indicating that almost all the retrogenes belong to different families in every species. This approximate one-to-one relationship is partially due to the stringent standards that we used to obtain the data. However, even without the restrictions, such as 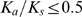 and different chromosomal locations between parental genes and retrogenes, the ratios in almost all species are still significantly less than 2 (Table S2).

**Table 1:** Statistics of retrogenes and retrofamilies.

Species	# of retrogenes	# of retrofamilies	# of retrogenes per family
Human	163 (163)	150	1.09
Chimp	199 (80∼139)	187	1.07
Macaca	275 (110∼193)	240	1.15
Mouse	154 (154)	144	1.07
Rat	226 (170∼203)	202	1.12
Dog	95 (71∼86)	90	1.06
Cow	163 (122∼147)	148	1.10
Opossum	232 (93∼162)	220	1.05
Chicken	99 (40∼69)	89	1.11
Zebrafish	140(105∼126)	122	1.15
Fruitfly	212 (212)	188	1.13
Anopheles	108 (43∼76)	101	1.07

The criteria for refining retrogenes for functionality vary among species. The numbers in the parenthesis are the estimated numbers of functional retrogenes. See Text S1 for details.

### Time distributions of retrogene pairs

**Figure 1:**
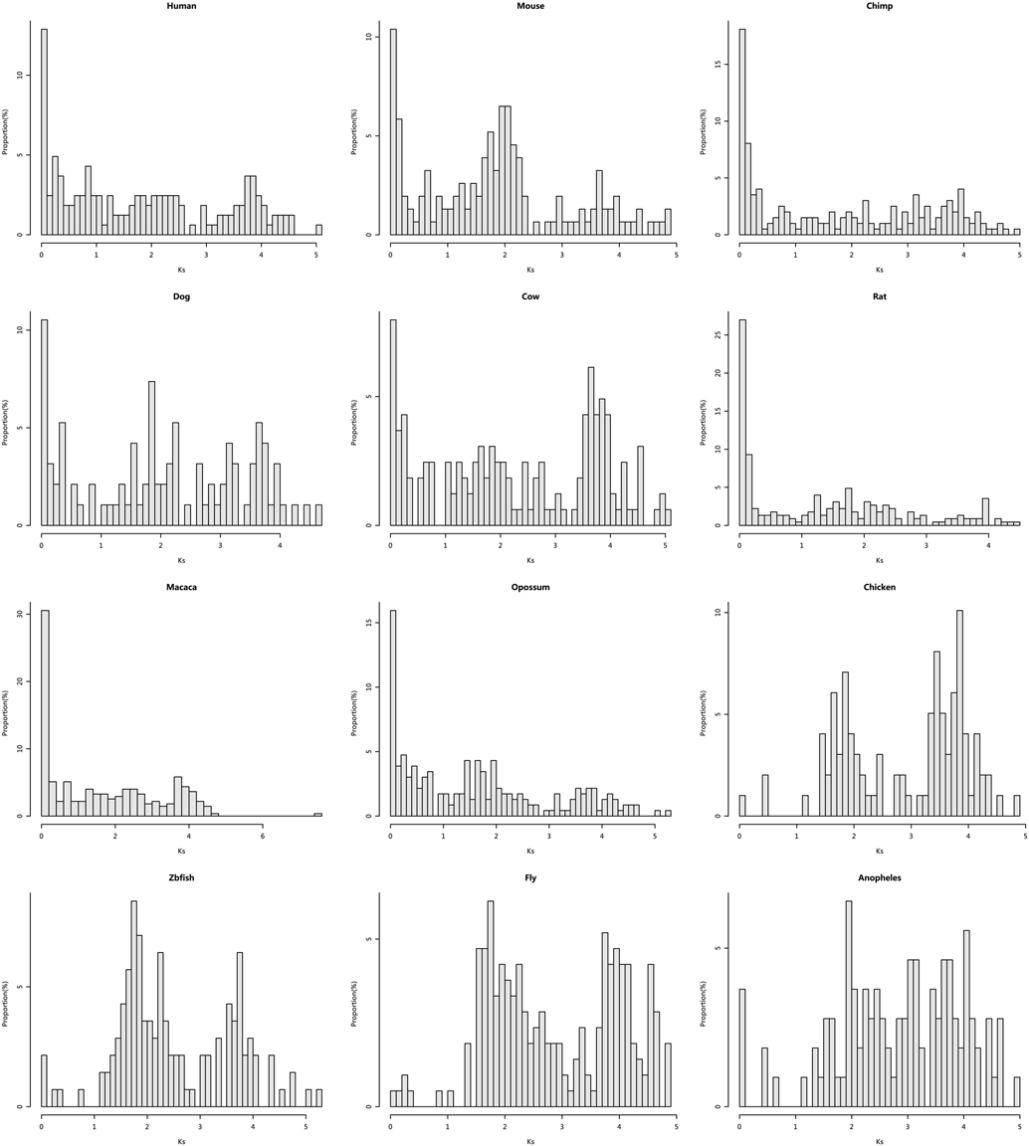
Distributions of 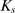 distances between parental genes and retrogenes.

To obtain a time distribution of retrogene formation events, we plotted the 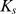 distributions of the parental-retrogene pairs for all species (Figure 1). Obviously, the 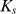 distributions between mammalian and non-mammalian species exhibit very different patterns. In mammals, there is a high proportion of retrogenes within small 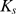 regions and at least about 10% of the parental-retrogene pairs have 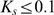. While in non-mammals, such pattern does not exist: less than about 3% have 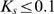 and the majority of parental-retrogene pairs are highly diverged (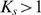). The burst of retrogenes in small 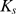 regions in mammals implies that a large number of retrogenes have occurred in mammalian lineages. As synonymous substitutions may be saturated for large 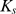, we also examined the 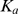 distributions. Results show that the distributions of 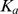 are similar to those of 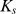: most mammals have the highest proportions of retrogenes in the small 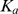 regions, while most non-mammals do not (Figure S1).

### Retrofamilies are shared between species

Since the number of retrogenes is approximately equal to the number of retrofamilies, we compared the retrofamilies across the species directly. We define the retrofamilies that are present in only one lineage as lineage specific retrofamilies (LSRs). Thus the non-LSR retrofamilies are shared by at least two lineages. Clearly, the number of LSRs in a certain lineage is mostly affected by its closest related lineage being compared. The higher the divergence between two species, the more LSRs we expect to see in each of the lineages.

**Figure 2:**
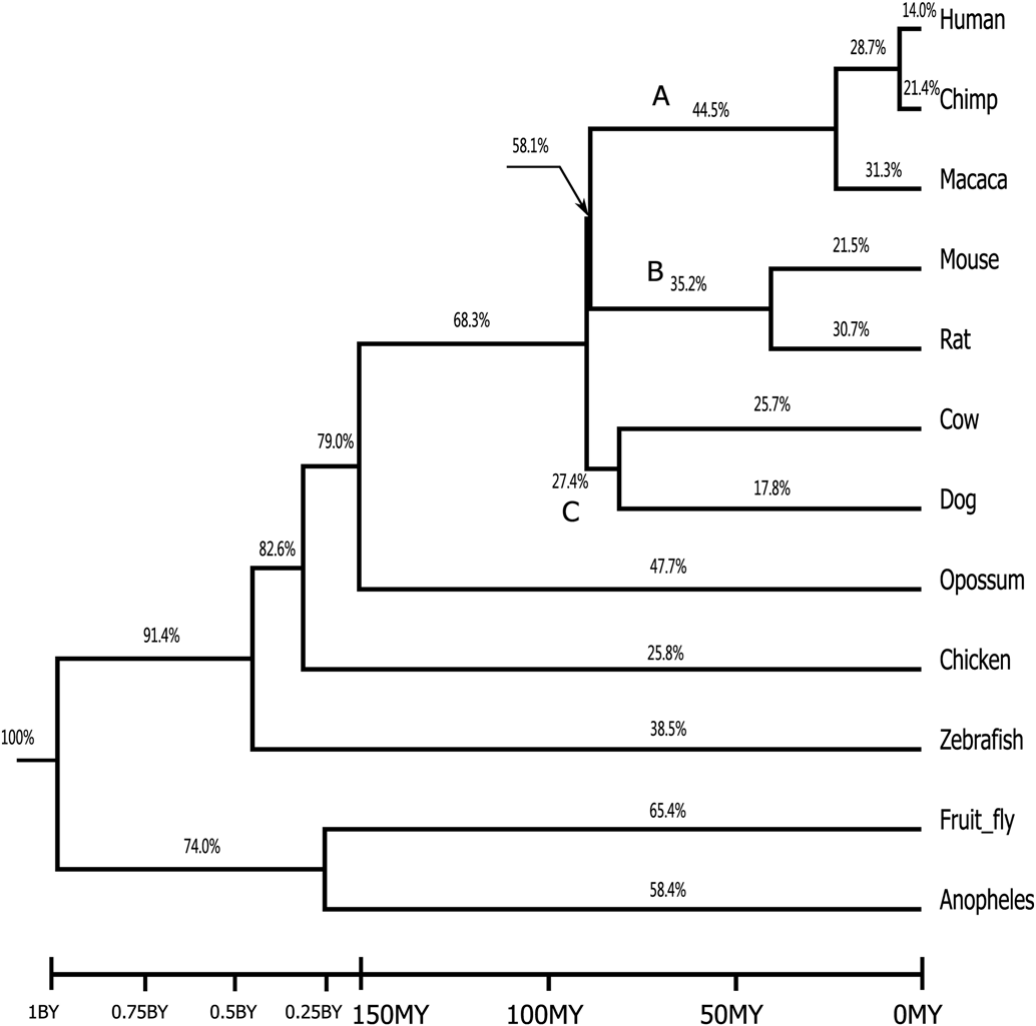
The species tree is adapted from Hedges [36]. The percentage of LSRs in a particular lineage (shown on each branch) is the ratio of the number of LSRs in the lineage to the total number of retrofamilies that the lineage has. Branch A is the primate lineage; Branch B is the murine lineage; Branch C contains dog and cattle.

We mapped the percentages of LSRs onto the species tree (Figure 2, see Table S3 for detailed retrofamily distribution). The percentage of LSRs in a particular lineage is calculated as the number of LSRs in the lineage divided by the total number of retrofamilies that the lineage has. For example, there are altogether 284 retrofamilies in the murine lineage (branch B in Figure 2), of which 100 are found only in murines (i.e. in mouse and/or rat), so the percentage of LSRs on branch B is 100/284 = 35.2%. The most prominent finding is that the percentages of LSRs on the external branches of all species except insects are less than 50%, and the proportion of LSRs in every mammalian species except opossum (about 44.3%) is no more than about 30%. It shows that more than 50% of the retrofamilies are not LSRs in mammals, suggesting that most of the retrofamilies in mammals are shared retrofamilies. The statement also holds even for some multiple-species lineages, such as the primate lineage (Branch A, 44.5%), the murine lineage (Branch B, 35.2%), and the lineage including cattle and dog (Branch C, 27.4%).

### Phylogenetic trees of mammalian retrofamilies

**Figure 3:**
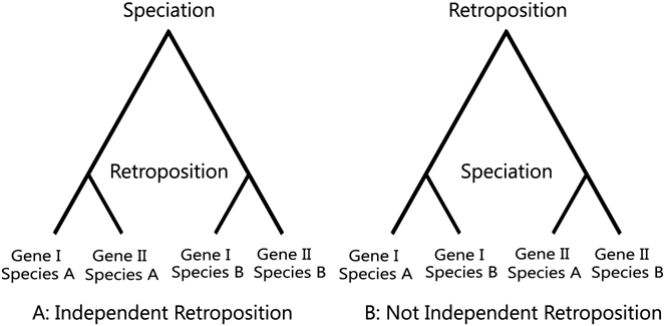
Illustration of independent and non-independent retroposition.

To test whether many retrogenes independently occurred in mammalian lineages, we constructed phylogenetic trees of parental genes and retrogenes in all shared retrofamilies of mammals and based on the tree topologies determined whether or not retroposition occurred independently in multiple mammals (see Materials and Methods for details). An independently-occurred shared retrofamily (IOSR) will have a tree topology similar to Figure 3A and a non-IOSR to Figure 3B. There are a total of 297 retrofamilies that are shared by at least 2 mammalian species. We obtained 296 trees and were unable to compute one tree due to high sequence divergence. Of the 296 trees, 57 trees follow strictly the pattern illustrated in Figure 3A. As human and chimp are closely related, we also considered the two species together as the great ape taxon and obtained 7 additional IOSRs. So, we have altogether 64 IOSRs out of the 296 retrofamilies, showing that about 22% retrogene formation events occurred in multiple mammalian species are independent (see File S1 for all IOSR trees).

### Functions of retrofamilies

To examine gene family functions of the mammalian retrofamilies, we obtained ENSEMBL family description for each retrofamily. Depending on the distribution of a retrofamily in different mammals, each retrofamily falls into one of the three categories: LSRs (the retrofamily is present in only one species), IOSRs (independently-occurred shared retrofamilies), and non-IOSRs. We found that the non-IOSRs do not show clear preference of certain functions. However, the IOSRs show a strong bias towards ribosome related gene families. About 28% ( = 18/64) of the IOSRs are ribosome related gene families, in contrast to the only 2.6% ( = 6/232) of the non-IOSRs retrofamilies that are ribosome related (Table S4). The exceptionally high proportion of ribosome related functions in IOSRs implies that ribosome related functions have the highest priority for retrogene formation in mammals. Interestingly, the preference for ribosome related function also exists in the LSRs of some species: 8%, 13%, and 17% of the LSRs in mouse, rat, and macaca respectively are also ribosome related, indicating that the emergence of ribosome related retrogenes may be a continuing process in mammals. In contrast, we found only one ribosome related IOSR in non-mammalian species. Moreover, only fruitfly and anopheles each have one ribosome related LSR. Thus, it seems that the high priority for ribosome related retrogene formation is a unique phenomenon in mammals.

## Discussion

Retroposition is an important process in generating new genes and functions [1]. Considering the mechanisms of retroposition, we hypothesized that there should be a recent retrogene burst in mammals not just in primates. We observed an unanimous pattern that supports our expectation from the 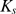 (and 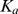) distributions of the retrogenes in all the mammals we studied. We also built phylogenetic trees to confirm that a large proportion of retrogenes occurred independently in mammals. Our observation confirmed our expectation that the fast acquirement of retrogenes is a common phenomenon in mammals rather than a special feature of the primate lineages [11].

### Burst of young retrogenes in mammals

The striking difference between mammals and non-mammals in the 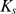 distribution is the presence of the 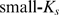 peaks in all studied mammals and the absence of them in all studied non-mammals (Figure 1). Why are there so many young retrogenes in these mammals? Several explanations can be made. First, the 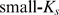 peak may be due to gene conversion between parental genes and retrogenes, which make old retrogenes appear young and thus inflate the proportion of retrogenes that have small 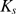 distance from their parental genes. However, this explanation is unlikely because the surveyed parental genes and retrogenes are on different chromosomes and gene conversion has been shown to be rare between genes on different chromosomes [23].

Second, the 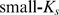 peak could be an artifact of the inclusion of many young intact but non-functional retrocopies. This issue is directly related to the criteria used to get retrogenes and their performance in ensuring retrogene functionality. As all retrogenes in human, mouse, and fruitfly have transcription and/or protein evidence for their functionality (see Table S5 for experimental evidence), plus the fact that we removed those retrogenes that have either no sequence divergence from or shared evidence with their parental genes, the patterns shown in these species should be highly reliable. In rat, cow, dog, and zebrafish, due to lack of experimental evidence, we had to include some predicted genes. However, the most conservative estimate of the probability of the retrocopies being a true retrogene in these species is still as high as 75% to 90% (Text S1) and applying this probability estimate to the small 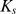 regions does not change the pattern qualitatively. For chimp, macaca, opossum, chicken, and anopheles, we used the computational criterion of 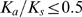 to refine the retrogene data. We estimated that about 30% to 60% genes that have 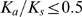 are likely non-functional (Text S1) and removing these proportion of genes in the small 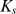 regions does not change the overall pattern. We also showed that the distributions of retrogenes are different from that of processed pseudogenes using human as an example (Text S1).

Taken together, both gene conversion and inclusion of non-functional retrocopies cannot explain the concordant pattern of 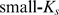 distributions in all the surveyed mammals. A third and more plausible explanation is the burst of young retrogenes in these mammals. This means that the recent quick formation of retrogenes is not a unique phenomenon in the primate lineage as Marques et al. [11] suggested, but a common phenomenon in at least the mammals that we studied. Therefore, it might not be seen as some kind of hallmark that contributes to merely the formation of human or primates.

The absence of parental-retrogene pairs with 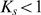 in the studied non-mammals suggests a lack of recent retrogene formation in these species. It has been suggested that the overall low level of retrogenes in the chicken is because the reverse transcriptases encoded by the unique LINE-like elements are unlikely to copy poly-A mRNA [24], [25], [26]. The fruitfly genome has a higher diversity of retroposons than the human, however, it seems to have a different response to the retroposons, and it has been generally accepted that the euchromatic retroposon inserts are deleterious and thus eliminated by strong purifying selection [27]. Thus, although the retroposition activity in the fruitfly seems to be much higher than that in the human, retroposon inserts in the fruitfly experience quick turnover (i.e., birth and death of retrotransposons). The dynamics of the retroposons in the zebrafish seems to resemble that of the fruitfly. Despite that the zebrafish has many different lineages of L1 (much greater diversity than mammals) [17], [28], the L1 copy numbers are controlled by strong purifying selection, which lead to high turnover rates of these elements. In anopheles, it has been shown that there have been rapid stochastic loss of some retrotransposons [29], but whether this is directly related to the lack of recent retrogenes needs further investigation.

One word of caution is that the lack of recent retrogene formation in the non-mammals rests upon the assumption that rates of synonymous substitutions in these non-mammals are similar to those in mammals. If the synonymous substitution rates in these non-mammals are 10 times or more faster than those in mammals, then the recent retrogene formation in mammals will correspond to the genes that fall in the 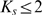 in the non-mammals. Rates of synonymous substitutions in different genes and different species vary a great deal (e.g. [30], [31]). It is difficult to quantify how much rate variation has contributed to the difference between mammals and non-mammals. As we also found similar pattern in 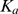 distributions and inter-species rate variation in 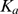 is not supposed to be large, we think the difference between mammals and non-mammals, for the most part, reflects real difference in retrogene dynamics between them.

### Independent formation of mammalian retrogenes

Most of the retrofamilies in mammals are shared by multiple mammalian species. One explanation for this observation is that the retropositions might have occurred in the ancestral lineage of the mammals that share the retrofamilies (non-independent retrogene formation). A second explanation is that retropositions of genes from the same families occurred independently in each lineage (independent retrogene formation). Also likely is a mixture of the two scenarios. These scenarios can be distinguished by the branching patterns of phylogenetic trees constructed with shared retrofamilies using the idea illustrated in Figure 3. The results show that about 22% ( = 64/296) of the shared retrofamilies have independent retrogene formation events in mammals. This is most likely an underestimate because we required that the parental-retrogene pairs in IOSRs follow strictly the pattern shown in Figure 3A, and if we relax this stringent criterion and include cases where retrogene formation events may have occurred independently in some but not all the species sharing the retrofamily, we will have more IOSRs. In addition, our method for identifying functional retrogenes in some species by limiting 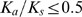 can lead to the exclusion of those retrogenes under weak purifying selection, neutral evolution, or positive selection. Therefore, the actual number or proportion of IOSRs should be higher than our current estimation.

Note that possible inclusion of processed pseudogenes only has a limited effect on the high occurrence of true IOSRs: with consideration of the likely inclusion of processed pseudogenes, we estimated that the expected number of true IOSRs is about 42 to 53 (Text S1), based on which, the final percentage of shared retrofamilies that have independent retrogene formation events in mammals is about 14%–18% ( = 42–55/296). Moreover, as processed pseudogenes evolve much faster than functional genes, it is less likely for them to cluster with their parental genes forming a topology strictly like Figure 3A except when they were born very recently. But the average 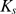 of mammalian retrogene pairs in all candidate IOSRs is as high as 0.49, indicating that recently born retrogenes (recently born retrocopies generally have a 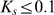 [14]) are not frequent in IOSRs.

Our results form a sharp contrast with the observation in *Drosophila* where only 3 (or 3%) independent retroposition events were found in 12 fly species [13]. The reason may be because the formation speed of retrocopies in primates (possibly also mammals) is twice that of *Drosophila* [11], [13]. The high rate of DNA loss in *Drosophila* may reduce the likelihood of retroposed copies to become real genes. It is likely that before the newly retroposed copy has a chance to recruit upstream regulatory elements, it might get deleted due to either the high rate of genome wide deletions or strong negative selection [32], [33], [34].

### Enrichment of ribosome related gene families in independently occurred retrofamilies

Interestingly, ribosome related gene families are enriched in the IOSRs of mammals (28%), but not in non-IOSRs of mammals (2.6%), nor in any types of retrofamilies of non-mammals. The exceptionally high proportion of ribosome related functions in IOSRs indicates that ribosome related functions have the highest priority for retrogene formation in mammals. However, as several thousand processed pseudogenes have been found in the mammalian genomes and nearly one fifth of them are ribosome related [5], the enrichment of ribosome related function in the IOSRs might be due to the inclusion of those intact but non-functional ribosome related retrocopies (or possibly processed pseudogenes).

If this is the case, taking human as an example, we can estimate quantitatively the effect of including ribosomal related non-functional retrocopies on the enrichment of ribosome related function in IOSRs. Since about 22.5% (1756/7819) of the processed pseudogenes are ribosome related [5] and among them about 12.3% (258/2090) are intact [3] (the numbers of ribosome related processed pseudogenes are slightly different between the two studies), the total expected percentage of intact non-functional ribosome related recopies is about 2.77%. Since IOSRs are shared by at least two species, we expect that the percentage of the ribosomal related retrogenes in IOSRs that are actually non-functional ranges from 0.077% (corresponding to the contamination of intact non-functional ribosome related retrocopies in both species) to 2.77% (corresponding to the contamination in one species). Taking into account this effect, we estimated that at least 27.6% ( = 18*(1–2.77%)/(64–18*2.77%)) of the retrogenes in IOSRs should be real and have ribosome related function, which differs little from the observed 28%. In fact, the actual proportion should be even higher as we did a rigorous functional assessment while compiling our dataset and our quantitative estimation shows that the influence of non-functional retrocopies is small (Text S1). Furthermore, most of the retrogenes in IOSRs have high 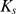 divergence from their parental genes, suggesting that possible inclusion of non-functional ribosome related young retrocopies contribute little to the enrichment of ribosome related functions in IOSRs. In addition, 8% of the retrogenes in mouse LSRs are also ribosome related. As the quality of the mouse data is very high, it leaves little room for doubting the presence of ribosome related retrogenes in mammals. Finally, if our observation is due to pseudogenes, the proportions of ribosome related genes in IOSRs and non-IOSRs should not differ by more than 10 folds. As ribosome related processed pseudogenes are widespread in mammalian genomes [3], [5], their occurrence rate in non-IOSRs should not be as low as observed. In fact, as aforementioned, our stringent way of identifying IOSRs will decrease the probability of including pseudogenes in IOSRs, despite which we still observe a high proportion of ribosome related retrogenes in IOSRs.

Demuth et al. [35] noticed that 18 out of 20 ribosome related gene family expansions in mammals are in the murine lineage. They proposed two hypotheses: the adaptive selection for increased reproductive rate and/or shorter generation time and the high rate of ribosomal protein retroposition with many intact but non-functional copies in rodent genomes. Our results show that the retroposition priority towards ribosome related gene families is not only present in the murine lineage, but also in other mammalian lineages. Thus, the increased reproductive rate and/or shorter generation time may be not the sole reason for the enrichment of ribosome retrogenes, especially in the lineages other than murines.

## Materials and Methods

### Datasets compiling

We studied eight mammals whose genomes have been assembled (not in scaffold stage) in ENSEMBL version 46 including human (*Homo sapiens*), chimp (*Pan troglodytes*), macaca(*Macaca mulatta*), mouse (*Mus musculus*), rat(*Rattus norvegicus*), dog(*Canis familiaris*), cow(*Bos taurus*), opossum(*Monodelphis domestica*), and four non-mammalian outgroup species including chicken(*Gallus gallus*), zebra fish (*Danio rerio*), fruitfly (*Drosophila melanogaster*), and anopheles(*Anopheles gambiae*). The phylogeny of these species is shown in Figure 2 (adapted from [36]). The opossum-eutheria divergence time (∼155 MY) was computed as the average of the divergence time estimates in several studies [37], [38], [39] and the fly-anopheles divergence time (∼250 MY) as in [40].

We retrieved the DNA and peptide sequences of all the species from ENSEMBL through BioMart [41]. To ensure annotation quality, we only used the genes whose chromosomal positions are known and peptides are longer than 50 amino acids. We used the longest transcripts for genes with multiple spliced forms. Then, we grouped genes into families using the ENSEMBL family annotation and paired genes within each family. ENSEMBL uses TribeMCL [42] a Markov clustering algorithm, to cluster all genes into families. It should be mentioned that ENSEMBL family IDs are not stable across versions and sometimes there are also minor changes to the contents of families. To make sure that our results are not influenced by different ENSEMBL versions, we also performed our analyses on two previous versions (v39 and v41) for both human and mouse and found that the results are very similar to results based on version 46.

We aligned the peptides of each gene pair using ClustalW [43]. To ensure valid homologous relationship, we discarded those pairs that have less than 70% amino acid overlap level. For the remaining gene pairs, we aligned DNA sequences using the peptide alignments as guidance and computed 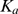 (the number of nonsynonymous substitutions per nonsynonymous site) and 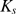 (the number of synonymous substitutions per synonymous site) by the YN00 program [44] in PAML version 4.0 [45].

### Retrogene screening

We retrieved ENSEMBL gene structure information. We used a two-step best hit method to screen the parental-retrogene pairs, similar to that used in previous studies [10], [11], [13]. The difference is that our method is based on the smallest synonymous divergence (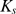) while all previous studies are based on the highest amino acid identity. First, since each retrogene can only have one parental gene, for each intronless gene, we chose the target gene that has the smallest 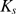 among all pairwise comparisons involving the intronless gene. If the target gene has multiple exons, we consider the target gene (parental gene) and intronless gene (retrogene) as a candidate parental-retrogene pair. We ignored those possible retropositions between intronless genes because they might not be generated by retroposition. Second, for each of the parental genes, we picked the retrogene that has the smallest 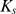 from its parent as the target retrogene. In this way, we ensured that the members of parental-retrogene pairs are mutual best hits of each other in terms of 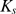. We also checked our result in the human with Marques et al. [11]. Most of their dated parental-retrogene pairs are also in our dataset. Only a few are different, all of which are due to the different versions of the human genome used in the two studies.

We discarded the pairs that are on the same chromosome to minimize the effect of gene conversion because gene conversion has been shown to be rare between duplicated genes on different chromosomes [23]. As about 80% of the parental-retrogene pairs are located on different chromosomes [14], only a handful of parental-retrogene pairs were removed.

### Functionality ensuring

To ensure functionality, we first removed the gene pairs with 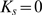. Because not all species are equally well annotated, we grouped species into three sets based on the availability of empirical evidence and applied a different standard to each group. For human, mouse, and fruitfly, we first obtained the possible Uniprot Unified Accessions and Uniprot Variant IDs for each protein coding gene from Ensembl, and then got all the PE (Protein Existence) status for those Uniprot Accessions or IDs. We required that all genes should have at least one UniProt entry whose PE evidence is annotated as “Evidence at protein level” or “Evidence at transcript level”. At the same time, we also required that the members within the same parent-retrogene pair should not share any experimental Uniprot entries. For rat, dog, cow, and zebrafish, we obtained the transcript status from Ensembl and required that all the transcripts of parental genes and retrogenes be annotated as “KNOWN”. For chimp, macaca, opossum, chicken, and anopheles, we required that parent-retrogene pairs should have 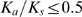. We also estimated the performance of these three criteria (see Text S1 for details).

### Phylogenetic analyses

We used programs in Philip version 3.6.1 [46] to construct the Neighbor-joining trees [47] with the F84 model [48], [49] for 1000 bootstraps for the retrogenes in shared retrofamilies. We used the Treegraph version 1.0 rc4 [50] to plot the trees.

We classified the trees into independently-occurred retrofamilies (IOSRs) and not-independently-occurred retrofamilies (non-IOSRs) using the idea illustrated in Figure 3. Suppose that one parental-retrogene pair (Gene I and Gene II) exists in species A and species B. Independent retrogene formation in the two species means that the speciation event predated the retrogene formation events and the retrogene formation events occurred independently in the two species. When free of gene conversion, the tree topology will be like Figure 3A. Otherwise, if retroposition occurred in the ancestor lineage, the tree topology will be like Figure 3B. Since our retrogene data is expected to be free from the influence of gene conversion, if the parental-retrogene pair in a species are clustered together before clustering with other species' genes and this is the case for all the species in the tree, we consider the retrogene an instance of independent retrogene formation in multiple mammals and call the retrofamily “independently-occurred shared retrofamily” (IOSR). We manually confirmed all the trees.

### Other data analyses

All the text parsing and processing procedures were done using a series of OCAML programs. Data were stored in a MySQL database for subsequent querying. All the statistical analyses were performed in R [51].

## Supporting Information

Text S1 (0.09 MB PDF)

Table S1 (0.51 MB XLS)

Table S2 (0.12 MB XLS)

Table S3 (0.23 MB XLS)

Table S4 (0.02 MB TAR)

Table S5 (0.21 MB XLS)

Figure S1 (0.69 MB PDF)

File S1 (5.25 MB TAR)
