# Supplementary material for: Burst of Young Retrogenes and Independent Retrogene Formation in Mammals
Source: PLoS One. 2009 Mar 27;4(3):e5040. doi: 10.1371/journal.pone.0005040 (PMC2657826; doi:10.1371/journal.pone.0005040)
Supplement: Text S1 — (0.09 MB PDF) [file pone.0005040.s001.pdf]

Supplement 1: Ensuring the Functionalities of Retrogenes

Deng Pan and Liqing Zhang

1 Three Functionality Criteria for Retrogenes

We defined *retrogene* as an "intact" retrocopy that has transcriptional evidence (see Results section). It is possible that some retrocopies are mis-annotated as retrogenes and included in our dataset. So, at first, we checked that all DNA sequences of candidate retrogenes have no frameshift mutations and no premature stop codons as compared to its parental gene, which ensures that all retrocopies in our study are "intact".

Next, we chose those "intact" retrocopies with experimental evidence as retrogenes. However, not all species have sufficient functional evidence for the putative retrogenes we identified. To deal with this difficulty, we further refined our data under three criteria with different stringency of functional evidence. During this process, we should ensure both high data quality (i.e. to minimize the influence of pseudogenes) and sufficient number of genes for analyses.

1.1 Criterion I

Criterion I is a widely-used computational criterion, which requires that parental-retrogene pairs have $K_a/K_s \leq 0.5$ (Betran et al., 2002; Emerson et al., 2004; Marques et al., 2005; Bai et al., 2007). This criterion has been discovered in fruitfly and confirmed in human. Betran et al. (2002) showed that 83% K_a/K_s of fruitfly's retrogene pairs are significantly smaller than 0.5. Marques et al. (2005) also showed that, in human, when $K_a/K_s \leq 0.5$, the proportions of processed pseudogenes are smaller than that of the intact retrocopies (see Figure 2 in (Marques et al., 2005)). The underlying theory of this criterion is that genes

with $K_a/K_s \leq 0.5$ are supposed to evolve under strong purifying selection and thus most likely have functions (Li, 1997).

Obviously, this criterion is not very stringent, and in fact, it is the least stringent criterion among the three. However, it does not require any experimental evidence and is applicable for all the species (Table 1). For the species that lack experimental evidence, such as chimp, macaca, opossum, chicken, and anopheles, we have to use this criterion. For other species, we can also use this criterion to compare with the data obtained from other criteria.

1.2 Criterion II

The second criterion requires that the transcript status of both parental genes and retrogenes should be annotated as "KNOWN" in ENSEMBL. According to Ensembl's annotation pipeline, only the transcripts that have entries in at least one of the three high quality databases: UniProt/Swiss-Prot, UniProt/TrEMBL, and NCBI RefSeq, have a "KNOWN" transcript status (http://www.ensembl.org/info/data/docs/genome_annotation.html). Thus, ENSEMBL claims that all genes under this criterion have experimental evidence.

However, this criterion can not guarantee 100% functionality because some "KNOWN" proteins in UniProt/Swiss-Prot, UniProt/TrEMBL or RefSeq are predicted, computed, or inferred from homology. Even though those predicting methods are based on the knowledge that eventually come from experimental information, it does not mean those predicted proteins themselves are 100% functional.

1.3 Criterion III

Since data passed *Criterion II* may contain predicted proteins, we further applied this stringent criterion by requiring that all genes should have at least one UniProt/Swiss-Prot or UniProt/TrEMBL entry that has a PE (Protein existence) line indicating that it has direct evidence at protein or transcript level (http://beta.uniprot.org/docs/userman.htm#PE_line). So, all "intact" retrocopies passed this criterion have direct experimental evidence, which confirms

100% to our definition of retrogene. We also removed those parental-retrogene pairs that shared experimental entries, which happens in some retrogene pairs with high sequence similarity. Since we will compare the three criteria, to be consistent, we also removed those pairs with shared evidence in the data under *Criterion I* and *Criterion II*. See Online Supplement file 9 for all the experimental evidence for human, mouse, and fruitfly.

Table 1 shows the number of retrogenes under the three criteria for all species. All retrogenes that satisfy *Criterion III* also satisfy *Criterion II* except fruitfly. For fruitfly, the "KNOWN"/"NOVEL" identification procedure is a bit different from the other species. The "KNOWN"/"NOVEL" status of fruitfly genes is set on the basis of whether the gene has a FlyBase symbol or not, rather than by UniProt or RefSeq evidence (personal communication with Ensembl). So, we ignored fruitfly's data under the *Criterion II*. Clearly, *Criterion III* is too stringent for most species except for human, mouse and fruitfly. To balance between data availability and reliability, we chose the retrogenes under the most stringent criterion, under which the number of retrogene is no less than half of that under the least stringent *Criterion I*, as the target dataset for each species in the following analyses (indicated by * in Table 1).

2 Performance of the Three Functionality Criteria

Since retrogenes satisfying *Criterion III* have direct protein and/or transcript evidence, the retrogene data under this criterion is of very high quality and can be used as the positive control for evaluating the performance of the other two criteria. Assuming that ENSEMBL gene annotation pipeline is not biased towards any particular species, the performance and the maximum type I errors ξ (i.e., the predicted retrogene is actually not functional–processed pseudogene) of the other two criteria should be similar across species.

Then, the probability P of a certain retrogene to be functional under the

three criteria will be

$$P(I) = (1 - \xi(I|II))(1 - \xi(II|III)) \tag{1}$$

$$P(II) = 1 - \xi(II|III) \tag{2}$$

$$P(III) = 1 \tag{3}$$

For $P(I)$, we used both $\xi(I|II)$ and $\xi(II|III)$, instead of $\xi(I|III)$ directly, because in this case, only three species (human, mouse and fruitfly) are used to determine $P(I)$. Our method used the information of almost all the species, and therefore should be less biased.

First, we estimated $\xi(I|II)$ by considering *Criterion I* vs. *Criterion II.* In this case, we used 6 species (human, mouse, rat,dog, cow and zebrafish) that have enough retrogenes under both criteria to do the evaluations. We grouped the parental-retrogene pairs under these two criteria in each species into three groups: Group A contains the pairs that satisfy *Criterion I* but not *Criterion II*; Group B contains the pairs that satisfy *Criterion II* but not *Criterion I*; Group S contains the pairs that satisfy both criteria (Table 2). So, $\xi(I|II)$ is calculated as the ratio of the number of retrogenes in A to the total number of retrogenes detected under *Criterion I*, which is about 20% - 45% in those surveyed species. Since retrogenes in Group A are not completely non-functional (they are removed just because we do not have any experimental evidence yet), the estimated $\xi(I|II)$ is therefore the upper limit of the true error rate.

Second, we considered *Criterion II* vs. *Criterion III* to estimate $\xi(II|III)$ At this stage, only human and mouse are available for comparisons. There are 117 and 52 retrogene pairs that have indirect experimental evidence in human and mouse (Table 1). These proteins are computationally predicted or inferred from homology. Since prediction methods should be better than random guessing and thus should have an accuracy rate higher than 50%, it is rather conservative to assume that only half of these predicted genes are true functional genes. Thus, we estimated the upper limit of $\xi(II|III)$ to be 13% for mouse and 21% for human. Therefore, for subsequent calculations, we used the range 10%-25% for $\xi(II|III)$.

Using the estimated $\xi(I|II)$ and $\xi(II|III)$ and equations 1 and 2, we estimated that $P(I)$ is around 0.4 to 0.7 and $P(II)$ is around 0.75 to 0.9. Note that in addition to requiring parental-retrogene pairs having $K_a/K_s \leq 0.5$ under the *Criterion I*, other research groups also did likelihood ratio tests and discarded the pairs that have a K_a/K_s not significantly less than 0.5 (Bai et al., 2007; Betran et al., 2002). However, we tested that if we used likelihood ratio tests, about 50% of retrogenes will be discarded. For example, if we used the likelihood ratio test, the number of retrogenes of fruitfly under *Criterion I* would be about 118, which is close to Bai et al. (2007)'s result (97). But, we think this method is too conservative because: first, still taking fruitfly for example, the number of retrogenes under *Criterion III*, which is the most reliable criterion, is much more than Bai et al. (2007)'s result; second, requiring K_a/K_s significantly less than 0.5 will remove the retrogene pairs whose K_a/K_s are slightly less or equal to 0.5, acorrding to the K_a/K_s distribution of intact retrocopies and retropseudogenes (Figure 2 in Marques et al. (2005)), which is about half of the number of total intact retrocopies ≤ 0.5 (note that under *Criterion I*, "intact retrocopy" is exchangeable with "retrogene"). In addition, since we used $P(I)$ to calibrate our numbers, we did not do the likelihood ratio test.

3 Comparing K_s Distributions between Retrogenes and Processed Pseudogenes

We further showed that the K_s distributions of retrogenes under the three criteria are different from that of the pseudo-retrogenes (Figure 1). The red line shows the distribution of retrocopies in the human genome (obtained from Figure 1 in Marques et al. (2005)), which contains both functional and nonfunctional retrocopies. Since intact retrocopies amount to only 18% of the retrocopies used in plotting the curve, and functional retrocopies should be even less than 18%, the red-line distribution should largely represent the K_s distribution of processed pseudogenes. If there is a serious contamination of processed pseudogenes in the retrogene datasets of all mammals, we expect

that the apex of the K_s distribution that contains pseudogenes be located at about $K_s = 0.1$, similar to what we see in the red-line distribution. However, none of the distributions under the three criteria confirm to this expectation. Instead, the apexes of the distributions of all our datasets are located in even smaller K_s regions. This phenomenon indicates that in contrast to processed pseudogenes which evolves much faster than functional genes, the enrichment of retrogenes in $K_s < 0.1$ regions is mainly due to functional retrogenes whose evolution is constrained purifying selection.

4 Estimating True IOSRs

If non-functional retrocopies are mistakenly annotated as retrogenes in some of the IOSRs, we will overestimate the number of retrogenes that were independently formed in multiple mammals. To quantitatively take account of this effect, we calculated the expected number of true IOSRs (N_t) using:

$$E(N_t) = \sum N_{mn} P(m) P(n) \tag{4}$$

where m or n means *Criterion* I, II or III. P is the likelihood that a retrocopy is a retrogene under a certain *Criterion*. N_{mn} is the number of IOSRs that are shared by at least two mammalian species under the most stringent criteria m and n. In this way, we obtained that within the observed 64 IOSRs, $E(N_t)$ is from 42.04 to 53.01, showing that at least 66% to 83% IOSRs are true IOSRs.

5 Tables

	# of retrogenes		
Species	*Criterion I*	*Criterion II*	*Criterion III*
Human	227	280	163 *
Chimp	199 *	20	1
Macaca	275*	4	2
Mouse	281	206	154 *
Rat	238	226 *	47
Dog	144	95 *	1
Cow	217	163 *	18
Opossum	232 *	2	1
Chicken	99 *	14	10
Zebrafish	165	140 *	40
FruitFly	237	57	212 *
Anopheles	108 *	13	2

Table 1: Number of retrocopies screened under the three criteria. * indicates the numbers that are used for final analyses.

Species	# of retrogenes			$\xi(I|II)$ (%) [a]
	Group A	Group S	Group B	
Human	50	177	103	22.0
Mouse	127	154	52	45.2
Rat	75	163	63	31.5
Dog	63	81	14	43.8
Cow	85	132	31	39.2
Zebrafish	45	120	20	27.3

Table 2: Comparison of the number of retrogenes under *Criterion I* and *Criterion II* in selected species. Group A contains retrogenes that satisfy *Criterion I* but not *Criterion II*, Group S contains retrogenes that satisfy both criteria, and Group B contains retrogenes that satisfy *Criterion II* but not *Criterion I*.
[a] $\xi(I|II)$ is calculated as $GroupA/(GroupA + GroupS)$.

Figure 1: Distributions of retrocopies under three criteria and the entire genome of human in small K_s regions. The red line indicates the proportion of retrocopies in terms of K_s in human obtained from Figure 1 in Marques et al. (2005), including both functional and non-functional retrocopies

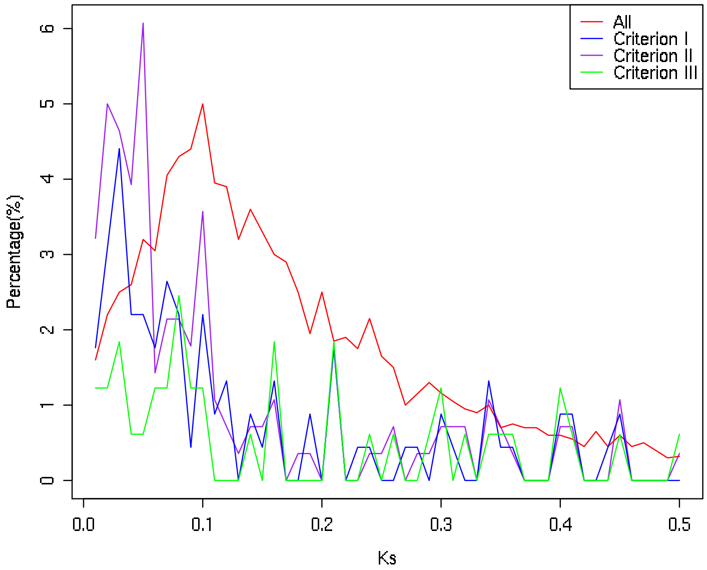

References

Bai, Y., C. Casola, C. Feschotte, and E. Betran (2007). Comparative Genomics Reveals a Constant Rate of Origination and Convergent Acquisition of Functional Retrogenes in Drosophila. *Genome Biol 8*(1), R11.

Betran, E., K. Thornton, and M. Long (2002). Retroposed new genes out of the X in Drosophila. *Genome Res 12*(12), 1854–9.

Emerson, J. J., H. Kaessmann, E. Betran, and M. Long (2004). Extensive gene traffic on the mammalian X chromosome. *Science 303*(5657), 537–40.

Li, W.-H. (1997). *Molecular evolution*. Sunderland, MA: Sinauer Associates.

Marques, A. C., I. Dupanloup, N. Vinckenbosch, A. Reymond, and H. Kaessmann (2005). Emergence of young human genes after a burst of retroposition in primates. *PLoS Biol 3*(11), e357.
