# Supplementary figures and images for: Burst of Young Retrogenes and Independent Retrogene Formation in Mammals
Source: PLoS One. 2009 Mar 27;4(3):e5040. doi: 10.1371/journal.pone.0005040 (PMC2657826; doi:10.1371/journal.pone.0005040)

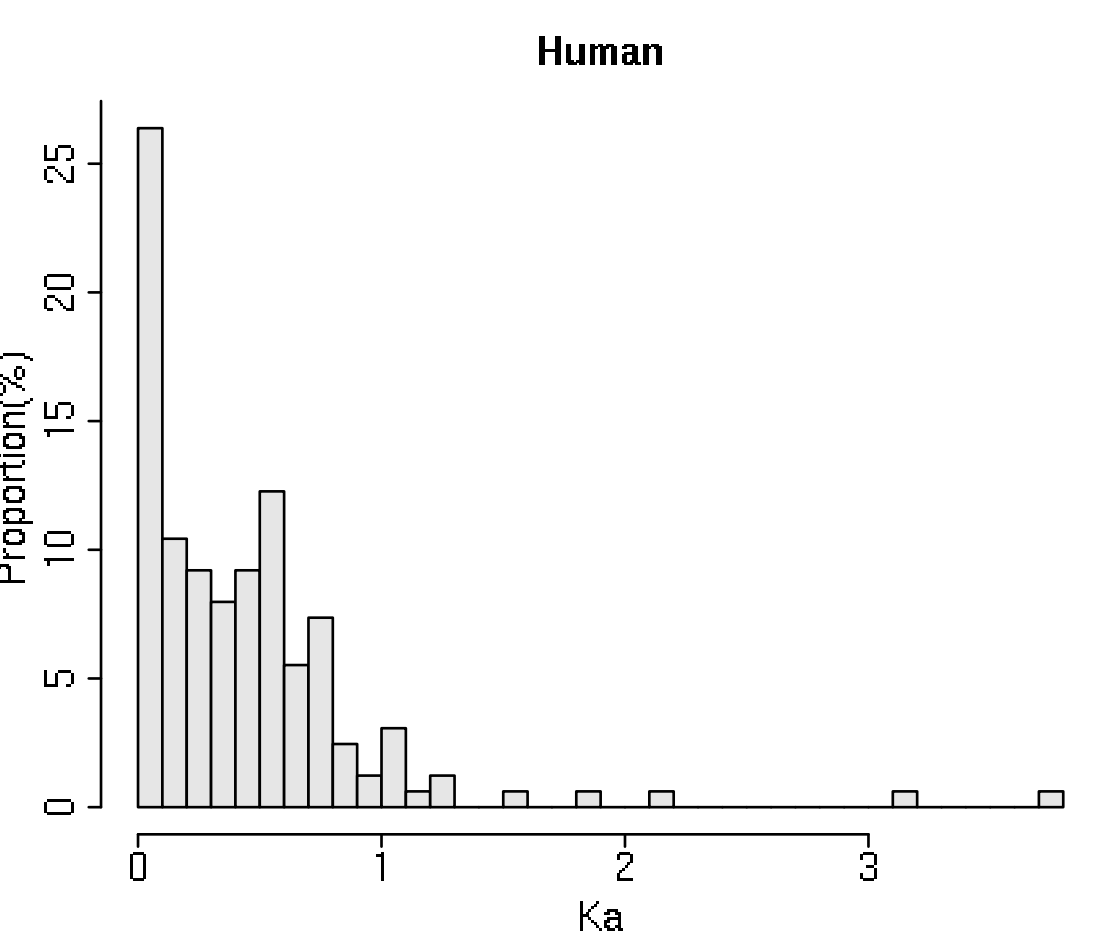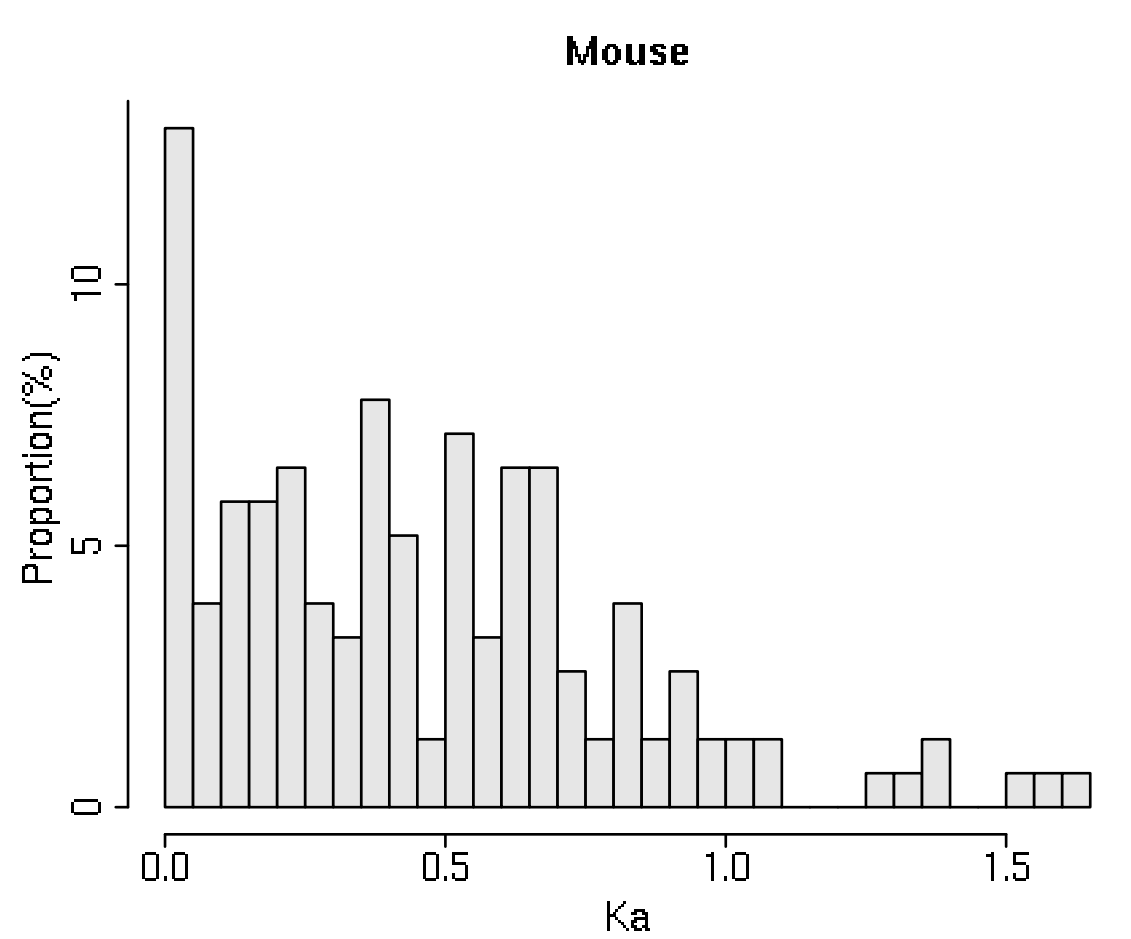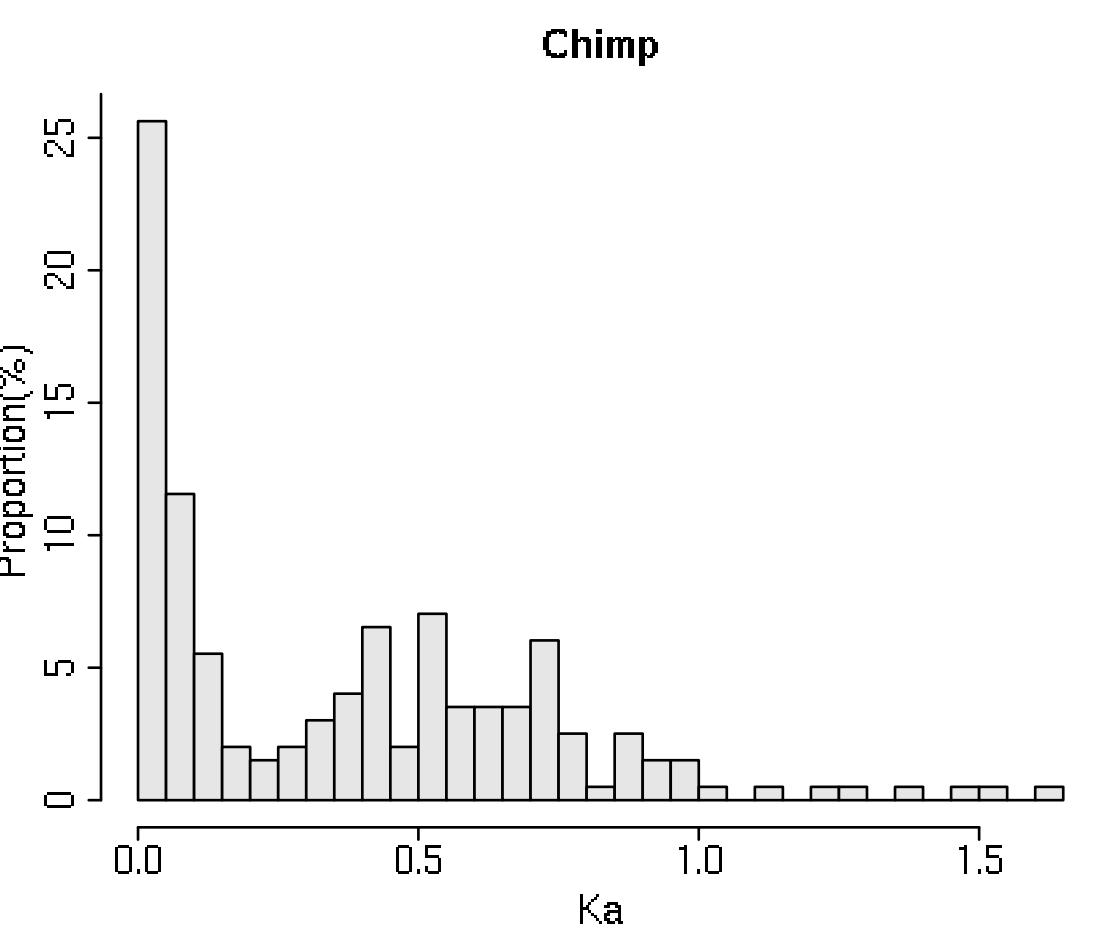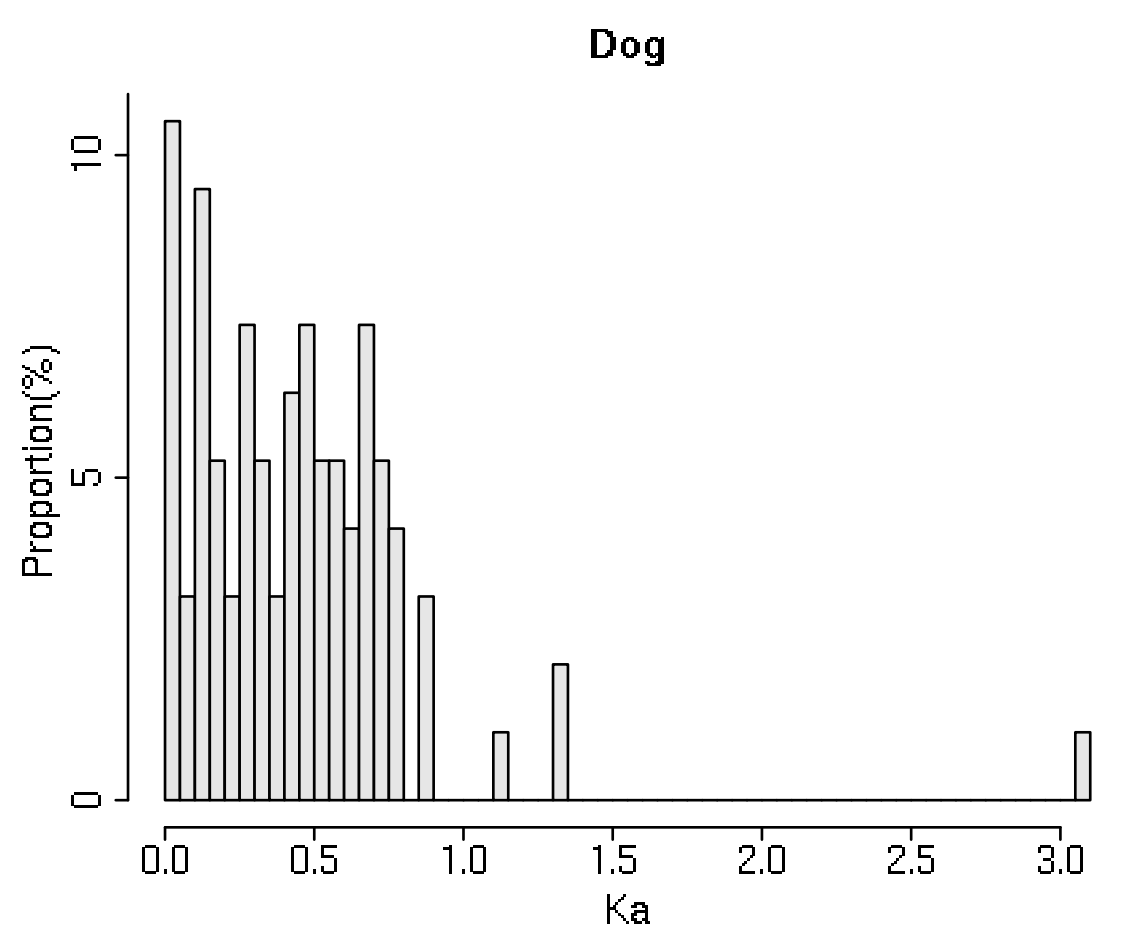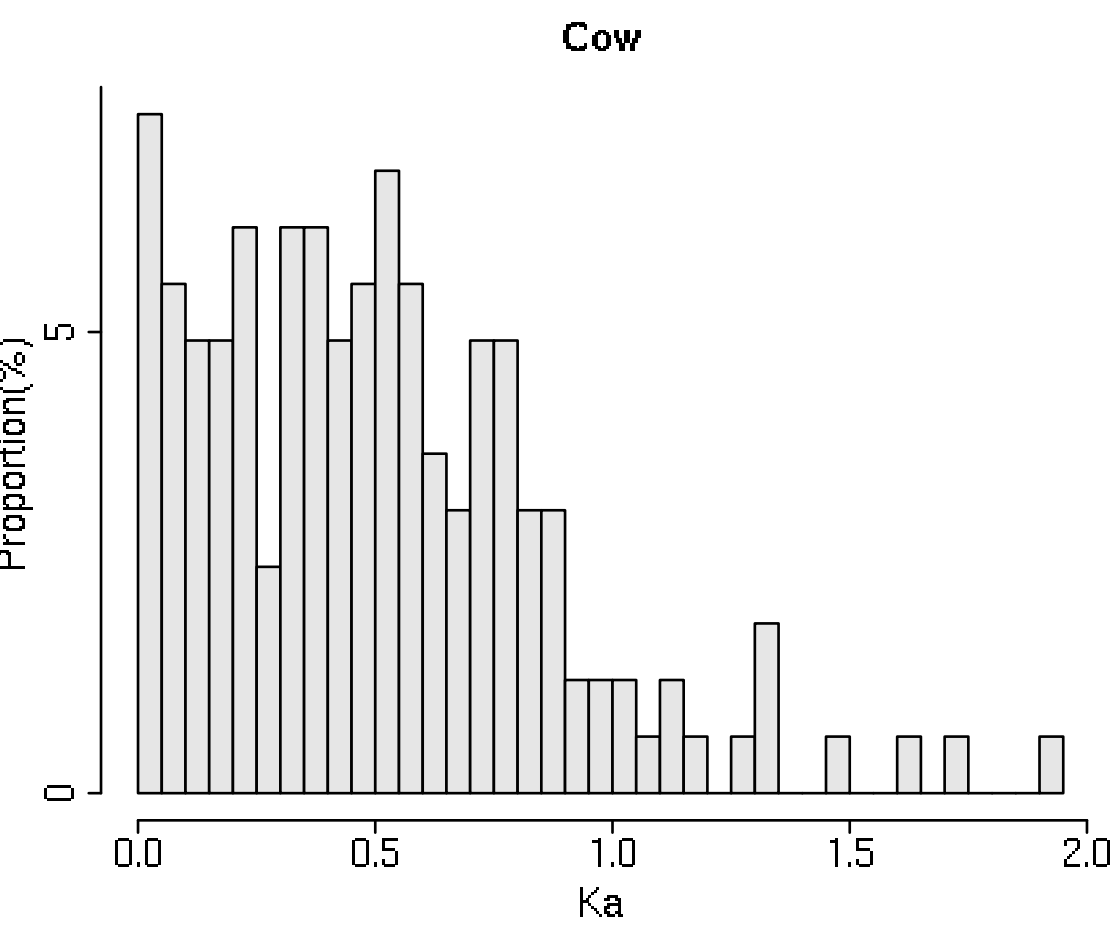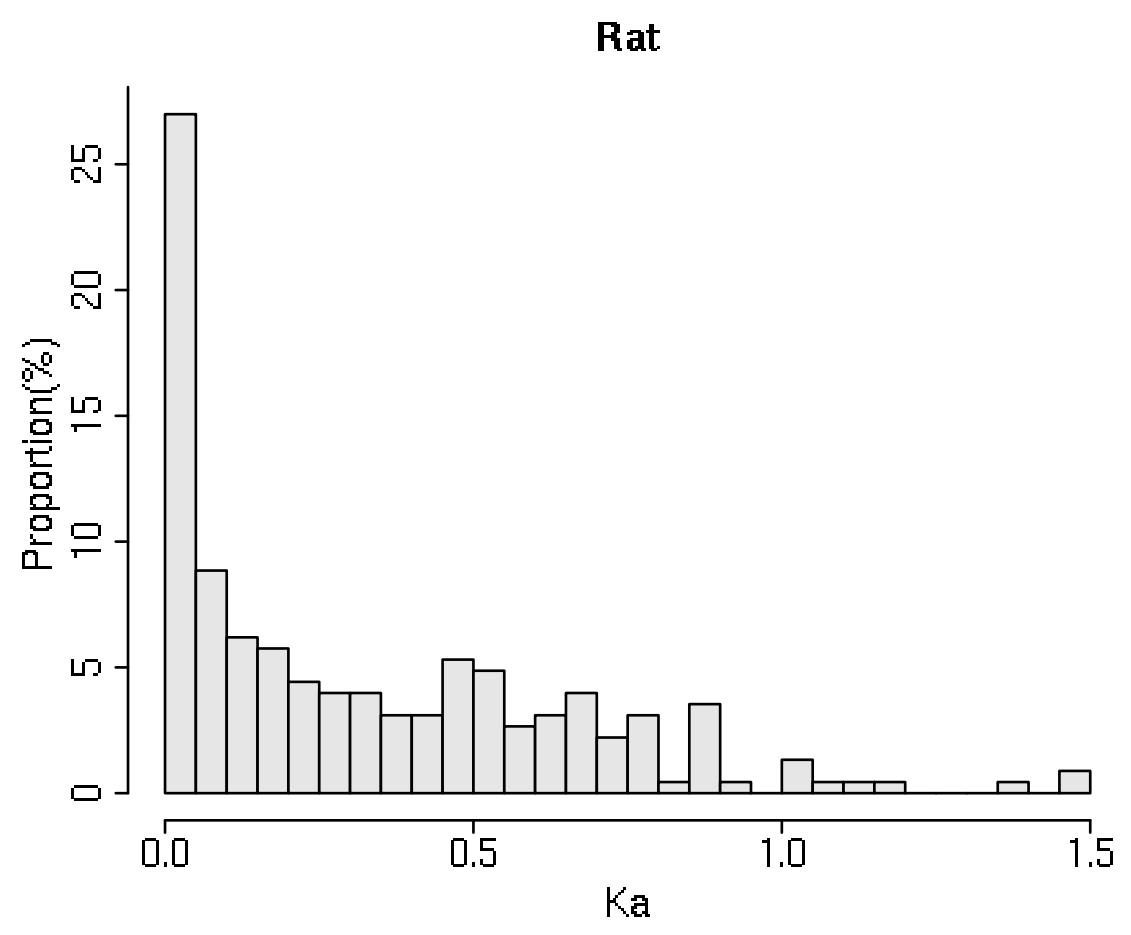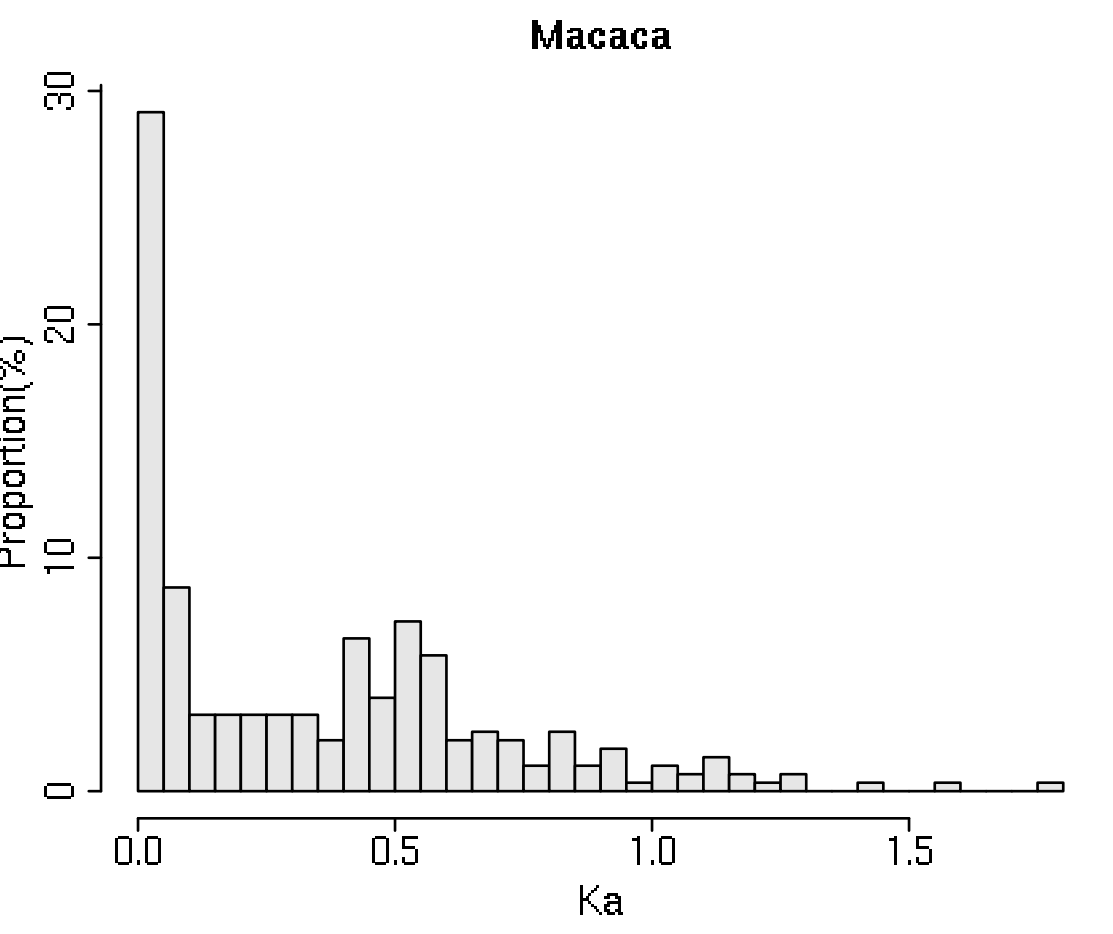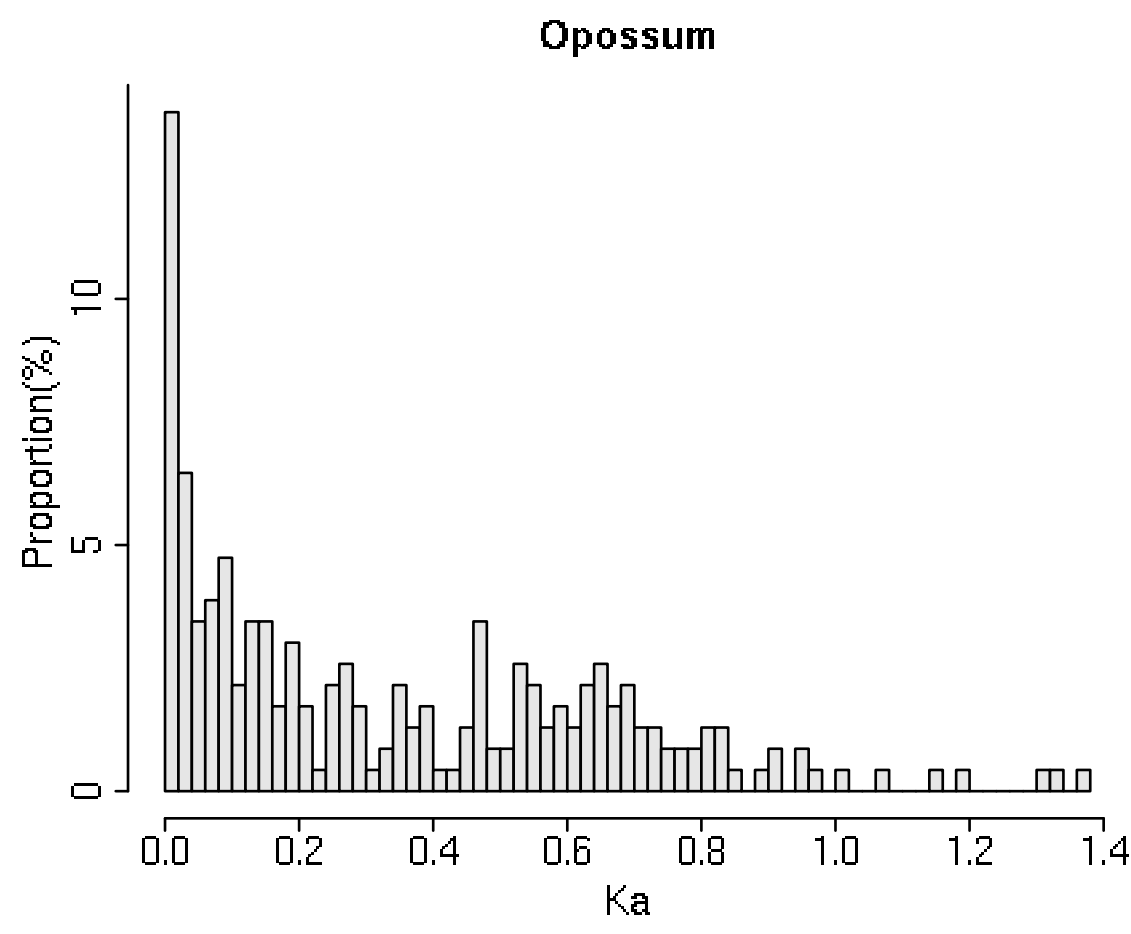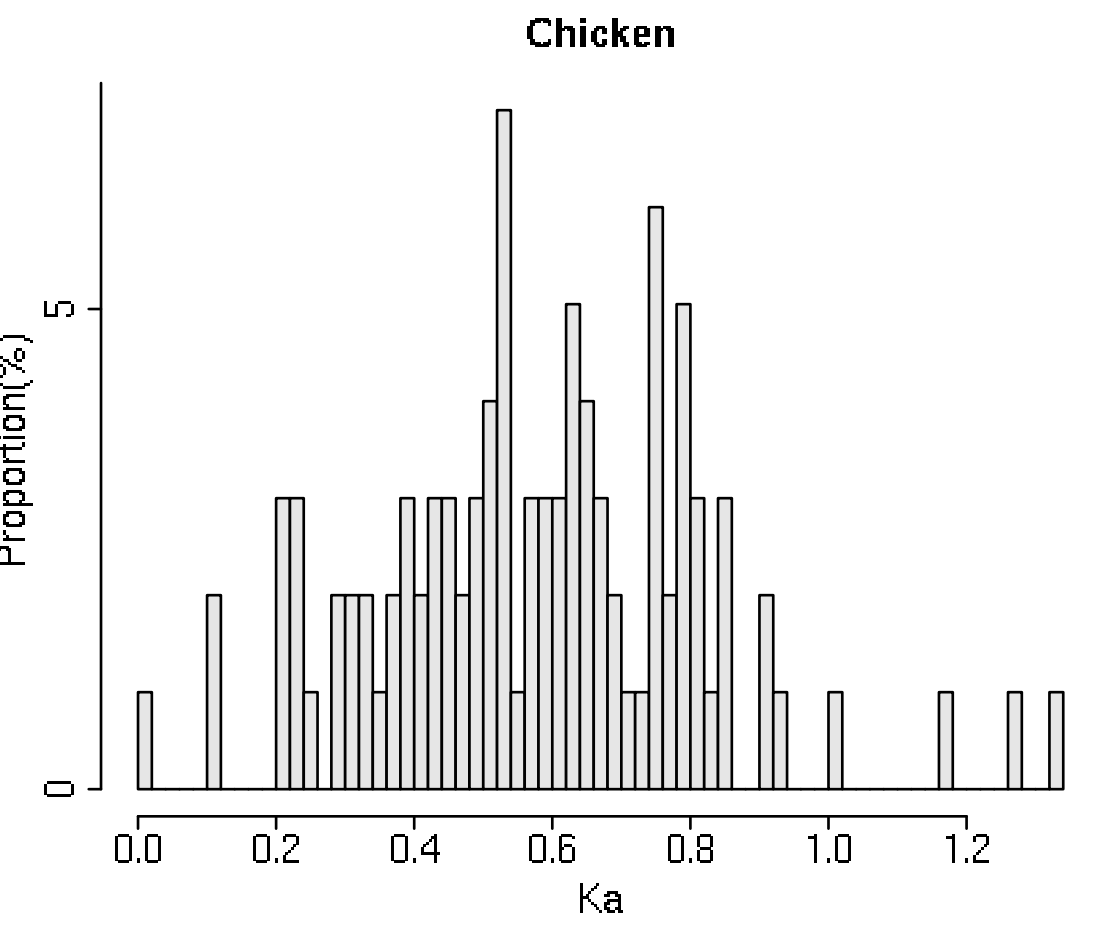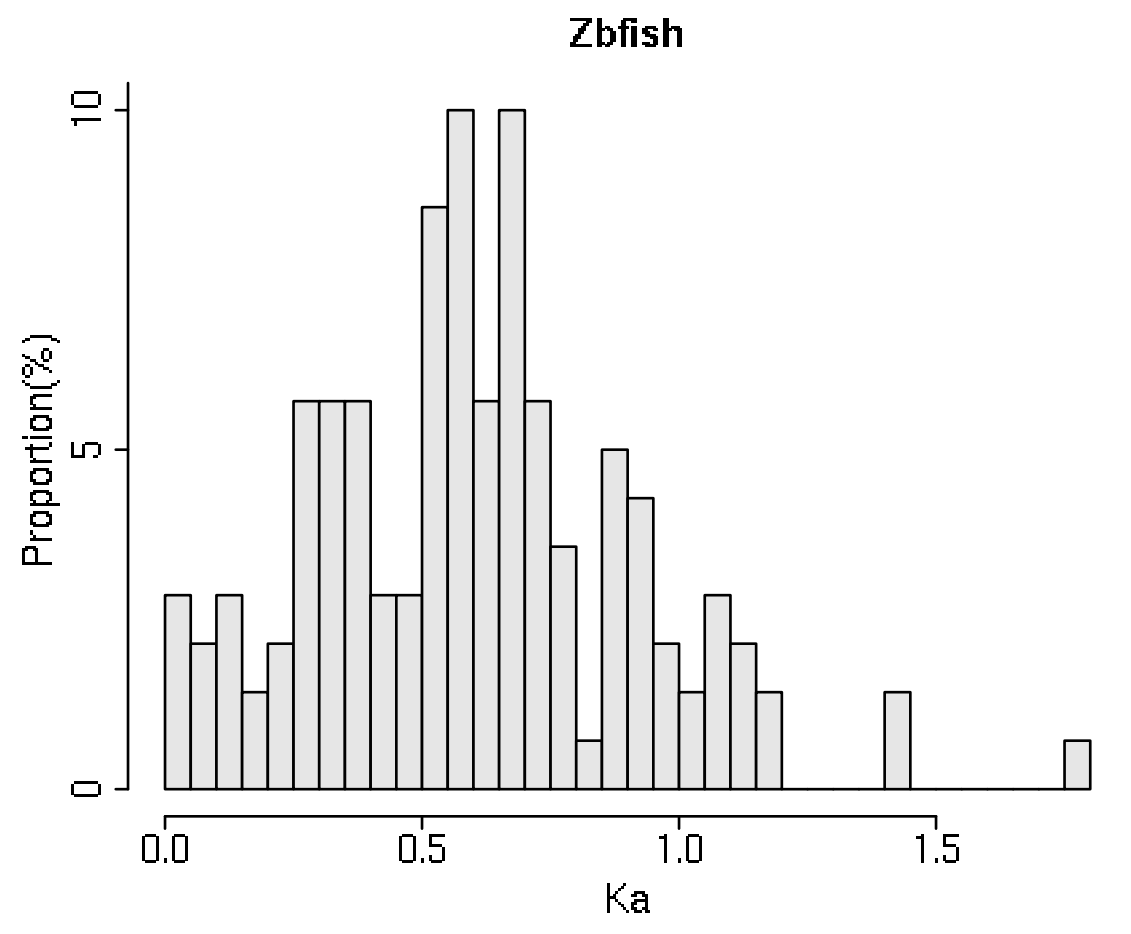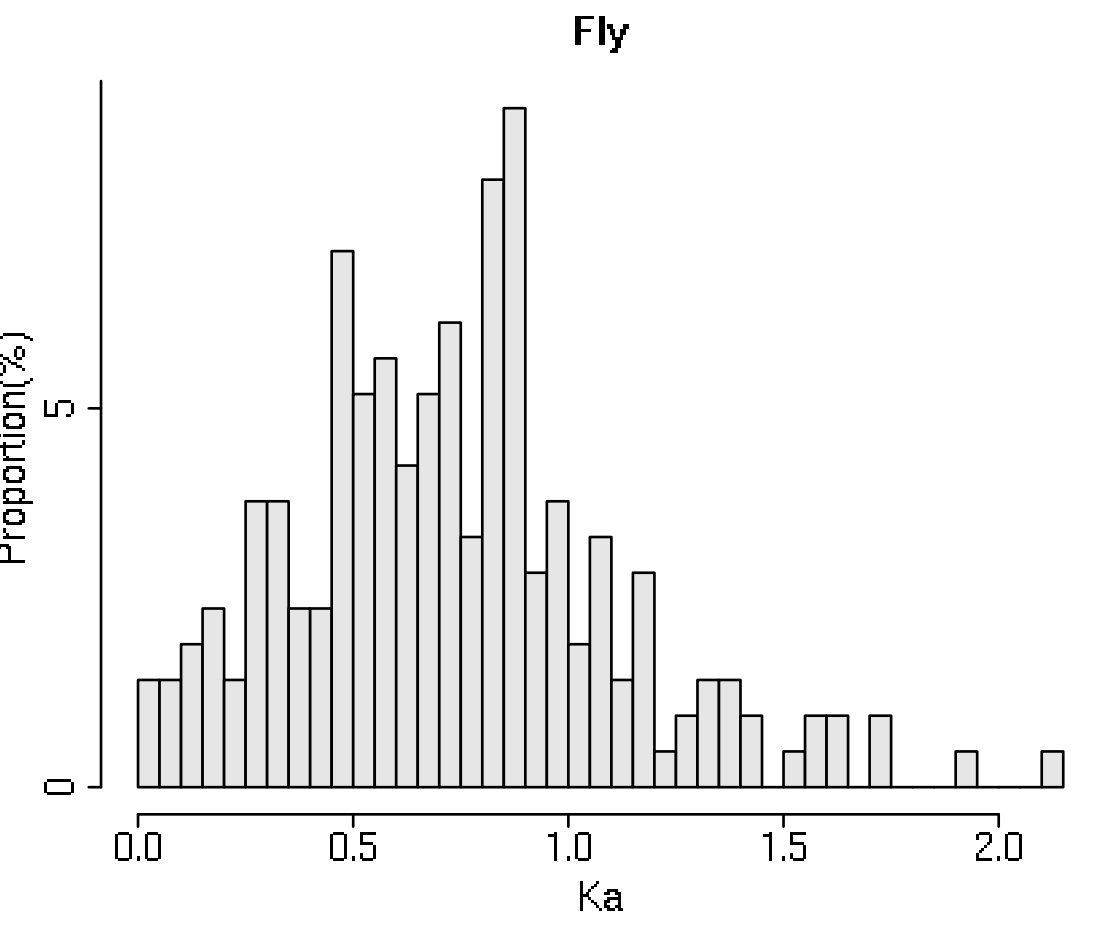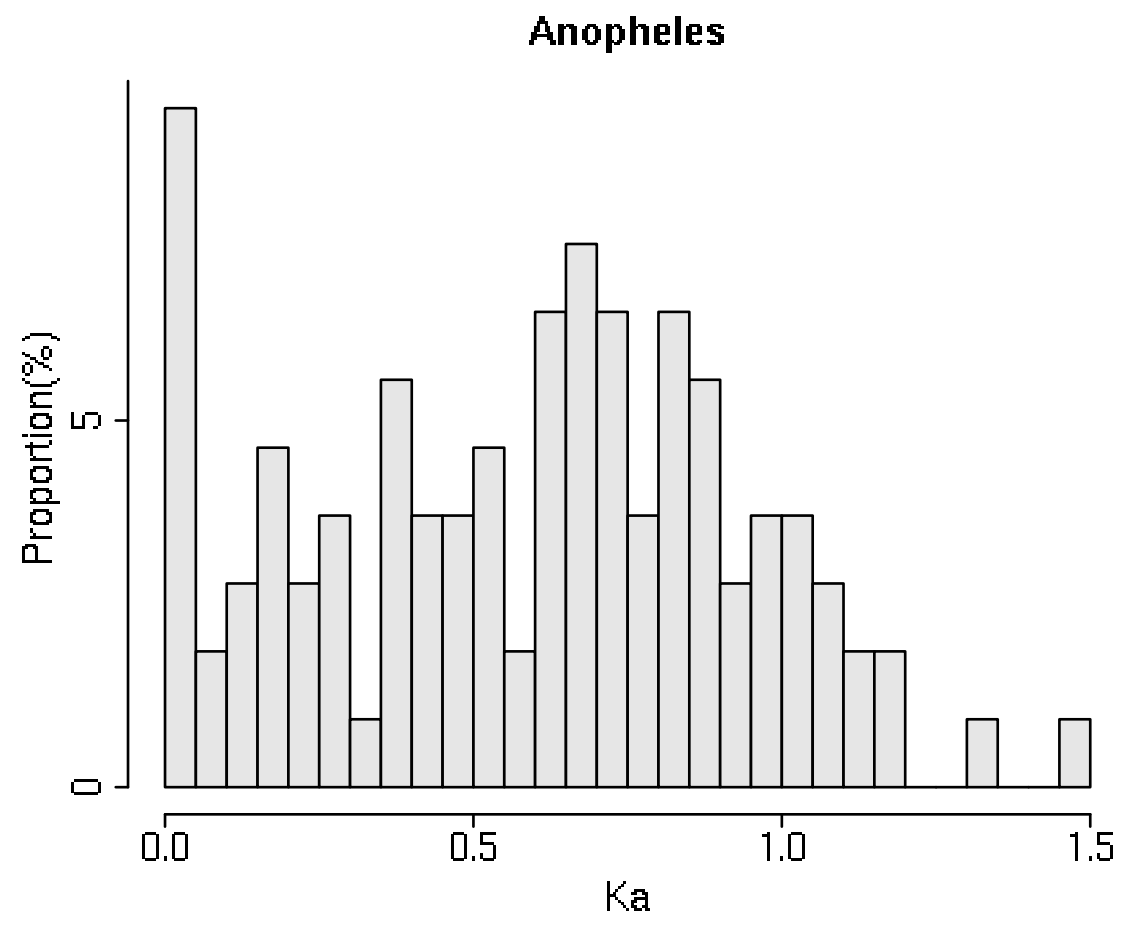

Supplement: Figure S1 — (0.69 MB PDF) [file pone.0005040.s007.pdf]
