# Supplementary material for: Burst of Young Retrogenes and Independent Retrogene Formation in Mammals
Source: PLoS One. 2009 Mar 27;4(3):e5040. doi: 10.1371/journal.pone.0005040 (PMC2657826; doi:10.1371/journal.pone.0005040)
Supplement: File S1 — (5.25 MB TAR) [file pone.0005040.s008.tar › iosrs/readme.doc]

Readme

1. Directory

high:	in including strictly mammalian IOSRS

hum_chimp: IOSRs formed by combining human and chimp as a complex taxon

2. each tree is named by Ensembl family id

3. Sequence information recovery

Since Phylip requires that the sequence name should be no more than 10 letters, we truncated and concatenated the original species names and Ensembl protein ids to 9 letters to generate the sequence names on the tree.

A seqence name contains two parts:

1) the first 3 letters are for species:
2) the last 6 letters are Ensembl protein id codes that need to be recovered by adding ensembl prefixes.

Here are detailed codes:

| Species | 3-letter code | prefix-for-last-6-letters |
| --- | --- | --- |
| human | hum | ENSP00000 |
| chimp | chp | ENSPTRP00000 |
| macaca | mac | ENSMMUP00000 |
| mouse | mou | ENSMUSP00000 |
| rat | rat | ENSRNOP00000 |
| dog | dog | ENSCAFP00000 |
| cow | cow | ENSBTAP00000 |
| opossum | opo | ENSMODP00000 |
| chicken | chk | ENSGALP00000 |
| zebra fish | zbf | ENSDARP00000 |
| Fruit fly | fly | CGXX (X are numbers) |
| Anopheles | mos | AGAP000 |

For example,

the original information of the sequence named “chk031966” on the tree is a chicken protein whose Ensemble id is ENSGALP00000031966

Note that, since the Ensembl Protein Ids for fly does not conform to this criterion, you need to find Ensembl Protein Id by searching the last 6 digit in the retrogene gene list.
